# Carrot Supplementation Improves Blood Pressure and Reduces Aortic Root Lesions in an Atherosclerosis-Prone Genetic Mouse Model

**DOI:** 10.3390/nu13041181

**Published:** 2021-04-02

**Authors:** Raffaella Soleti, Marine Coué, Charlotte Trenteseaux, Gregory Hilairet, Lionel Fizanne, Fatima Kasbi-Chadli, Patricia Mallegol, Julien Chaigneau, Jerome Boursier, Michel Krempf, Emmanuel Geoffriau, Ramaroson Andriantsitohaina, Khadija Ouguerram

**Affiliations:** 1 SOPAM, U1063, Inserm, SFR Interactions Cellulaires et Applications Thérapeutiques Université D’Angers, 49 933 Angers, France; raffaella.soleti@univ-angers.fr (R.S.); gregory.hilairet@univ-angers.fr (G.H.); patricia.mallegol@univ-angers.fr (P.M.); 2 UMR 1280 Physiopathologie des Adaptations Nutritionnelles (PhAN), INRAE, Université de Nantes, 44 093 Nantes, France; marine.coue@chu-poitiers.fr (M.C.); charlotte.trenteseaux@gmail.com (C.T.); Fatima.Chadli-Kasbi@univ-nantes.fr (F.K.-C.); Michel.Krempf@univ-nantes.fr (M.K.); 3 Centre de Recherche en Nutrition Humaine Ouest, 44 093 Nantes, France; 4 EA 3859, Hémodynamique, Interaction Fibrose et Invasivité Tumorales Hépatiques (HIFIH), Université d’Angers, 49 933 Angers, France; lionel.fizanne@univ-angers.fr (L.F.); julien.chaigneau@univ-angers.fr (J.C.); JeBoursier@chu-angers.fr (J.B.); 5 Institut Agro, Université d’Angers, INRAE, IRHS, SFR QUASAV, 49 045 Angers, France; emmanuel.geoffriau@agrocampus-ouest.fr

**Keywords:** carrot supplementation, hemodynamic parameter, atherosclerosis, high fat diet, ApoE^−/−^

## Abstract

Epidemiological studies have shown that carrot consumption may be associated with a lower risk of developing several metabolic dysfunctions. Our group previously determined that the Bolero (Bo) carrot variety exhibited vascular and hepatic tropism using cellular models of cardiometabolic diseases. The present study evaluated the potential metabolic and cardiovascular protective effect of Bo, grown under two conditions (standard and biotic stress conditions (BoBS)), in apolipoprotein E-knockout (ApoE^−/−^) mice fed with high fat diet (HFD). Effects on metabolic/hemodynamic parameters and on atherosclerotic lesions have been assessed. Both Bo and BoBS decreased plasma triglyceride and expression levels of genes implicated in hepatic *de novo* lipogenesis and lipid oxidation. BoBS supplementation decreased body weight gain, secretion of very-low-density lipoprotein, and increased cecal propionate content. Interestingly, Bo and BoBS supplementation improved hemodynamic parameters by decreasing systolic, diastolic, and mean blood pressure. Moreover, Bo improved cardiac output. Finally, Bo and BoBS substantially reduced the aortic root lesion area. These results showed that Bo and BoBS enriched diets corrected most of the metabolic and cardiovascular disorders in an atherosclerosis-prone genetic mouse model and may therefore represent an interesting nutritional approach for the prevention of cardiovascular diseases.

## 1. Introduction

Many epidemiological studies reported that diets enriched in fruits and vegetables reduced cardiovascular risk and metabolic disorders. In this context, carrot root represents an attractive healthy food in preventive nutrition as it contains bioactive compounds including carotenoids, vitamins, polyphenols, fiber, and minerals [1,2]. Interestingly, it has been shown that some of these carrot bioactive compounds had beneficial cardiovascular and metabolic properties.

Carrot antioxidant compounds including carotenoids and polyphenols are able to scavenge reactive oxygen species and increase endogenous defense systems resulting in a reduction of oxidative stress, overall decreasing the risk of cardiovascular diseases [3,4]. The high content of carotenoids and fibers contained in carrots acts as an antioxidant defense enhancer [5]. It has also been shown that carrot consumption, through the synergistic effect of fiber and antioxidant components, could induce a decrease in circulating lipids and improved antioxidant status reducing cardiovascular risks in cholesterol-fed mice [2]. Moreover, in a rat model of metabolic syndrome, purple carrot juice improved cardiovascular and hepatic structure and function as well as associated metabolic parameters, such as abdominal fat deposition and plasma lipid profiles [6]. Clinical studies have also shown that drinking carrot juice could protect the cardiovascular system by increasing the antioxidant status and decreasing lipid peroxidation regardless of the presence of risk factor [7].

Using a multiparametric screening on cellular models of cardiometabolic diseases, we have previously determined that carrot genotypes contrasted by root color and grown under different conditions displayed different pharmacological profiles on vascular and metabolic cells [8]. We have also reported that all assayed carrot extracts affected vascular cell oxidative stress and apoptosis, as well as metabolic cell oxidative stress and lipid accumulation. In the same study, we also found that carrots grown under biotic stress—induced by *Alternaria dauci* inoculation—were the more efficient in inducing beneficial effects on vascular and hepatocyte functions, with the Bolero (Bo) variety being the most effective [8]. However, the pathophysiological relevance of the effects of Bo in a model of lifestyle-related cardiometabolic diseases has not been investigated yet.

Thus, the present study evaluated the effect of Bo grown under two conditions (standard conditions (Bo) and biotic stress conditions (BoBS) induced by *Alternaria dauci* inoculation) in apolipoprotein E-deficient (ApoE^−/−^) mice fed with high fat diet (HFD). The effects on body weight, glucose and lipid regulation, hepatic, cardiac, and hemodynamic parameters—as well as atherosclerotic lesions—were evaluated.

## 2. Materials and Methods

### 2.1. Products

Plants of Bo carrot genotype were grown under a semi-controlled environment in tunnel in two biological repetitions in 2013 at Agrocampus-Ouest (IRHS-Angers, France). Carrots were grown (i) in standard growing conditions (no inoculation, Bo) or (ii) in biotic stress conditions with *Alternaria dauci* inoculation (BoBS), as previously described [9]. Carrot roots were taken grinded in bulk and snap frozen at −80 °C.

Incorporation of carrot samples at 25 g/kg in HFD deficient in choline was prepared by SAFE (Augy, France) and delivered in lyophilized form. Table 1 provides the nutritional composition and energy characteristic of diets.

### 2.2. Ethics Statement

All animal protocols were performed in animal facilities of Universities of Nantes and Angers. Experimental procedures were approved by the local ethics committee “Comité d’éthique en experimentation animale Pays de la Loire”; (Project No. Apafis#1687-2015060312546655 and Apafis#320-2015031314466612_v2) and realized in agreement with the guidelines and authorization with French Ministry of Agriculture regulations according to the European Union regulations (2010/63/EU).

### 2.3. Animals

Apolipoprotein E-deficient C57BL/6.129P2-APOE/J (ApoE^−/−^) mice of the different experimental groups were obtained by cross-breeding in the two laboratories (UMR1280 and U1063). In order to have a sufficient number of individuals for a good statistical power while respecting the 3Rs rule, imposed by the ethics committee, we used—for certain measurements—males and females, as well as the total number.

Female and male ApoE^−/−^ mice at eight-week-old were supplied from Charles River (L’Arbresle, France) and from the animal housing unit of the University of Nantes (Nantes, France) and the University of Angers (Angers, France). As the results obtained in male and female mice were similar, the results from both sex genders were grouped to gain statistical power for some of the performed analysis. The animals were housed in a controlled environment room with constant 12 h/12 h light/dark cycles, ambient temperature at 23 ± 2 °C, and had ad libitum access to water and food. Three groups of mice were used: HFD (data concerning these mice have been previously published by Soleti et al. [8]), and HFD containing 25 g of carrot without (Bo) or with biotic stress (BoBS)/kg for a period of 14 weeks.

Mouse body weight was weekly reported and diastolic and systolic blood pressure as well as heart rate were measured at four periods of the experimental design (before the introduction of the diet and after 1, 2, and 3.5 months), as previously described [8]. The day before the killing, echocardiography measurements were assessed. At the end of the protocol, mice were submitted to an overnight fasting (≈12 h) and then euthanized to collect blood, adipose tissues, liver, heart, and aorta for biochemical and histological analysis. Organs and tissues were frozen for further analysis.

### 2.4. Cardiovascular Parameters Measurements

To measure systolic, diastolic, mean blood pressure, and heart rate we used a non-invasive tail-cuff method as previously described [8]. Mice were used to wear the tail-cuff every day during five consecutive days to get accustomed to the device before the evaluation of baseline blood pressure corresponding to the average of ten successive measurements.

### 2.5. Echocardiography

Functional and structural heart modifications were evaluated by transthoracic echocardiography using the Vevo 770 ultrasound echography as previously described [8].

### 2.6. Biochemical Parameters

Fasting glucose, triglycerides (TG), total cholesterol, low density lipoprotein (LDL)-cholesterol, and high-density lipoprotein (HDL)-cholesterol were determined in plasma samples using Konelab™ 20 Clinical Chemistry Analyzer (Thermo Scientific™, Waltham, MA, USA). Enzyme-linked immunosorbent assay (Merck-Millipore, Darmstadt, Germany) were used to measure plasma insulin.

### 2.7. Hepatic Secretion of Triglycerides

To estimate hepatic TG production rate, Triton-1339 (Sigma-Aldrich, St Quentin Fallavier, France), an inhibitor of lipoprotein lipase, was intraperitoneally injected in mice at dose of 500 mg/kg of body weight [10]. Blood was taken from the tail vein at baseline and at 120 and 300 min. An enzymatic assay from Dyasis (Dyasis, Grabels France) was used to quantify TG concentrations. TG production rate, expressed as mg/dL/h, was therefore calculated, for each animal as previously described [11].

### 2.8. Liver Histology

The quantification of area of steatosis and fibrosis was assessed in lobe one and two slices as previously described [8].

### 2.9. RNA Extraction and Real-Time RT-qPCR

Tissue RNA extraction was realized using TRizol reagent (Life Technologies, Saint Aubin, France) following the manufacturer instructions. After DNAse treatment (Promega, Charbonnières-les-Bains, France) and reverse transcription of 1 µg total RNA processed, fluorescence of samples from SYBR Green qPCR were detected as previously described [8]. All primer sequences, ordered from Eurofins Genomics (Eurofins, Nantes, France), were available on request. All cDNA amplification were performed in triplicate, and Cyclophilin was used as an internal control. Expression data were normalized by the 2 (DCt) method using Tata-box binding protein (Tbp) as internal control.

### 2.10. Short Chain Fatty Acids Analysis and Quantification by Gas Liquid Chromatography

Cecal were collected for short chain fatty acids (SCFA) analysis and processed as previously described [12]. SCFA (acetate, propionate, butyrate) were analyzed by capillary gas liquid chromatography (SGE BP21 capillary column). Samples (1 µL) were introduced by splitless injection, with a split flow of 50 mL/min beginning 1 min after injection. The concentration of SCFA was determined by using a standard curve.

### 2.11. Atherosclerotic Lesions Analysis

Atherosclerosis progression were measured at on euthanized mice at 22 weeks old (*n* = 6–7 per group). The entire aorta between the heart and iliac bifurcation was carefully dissected and snap frozen at −80 °C. Aorta linked to the heart was later defrosted for microdissection and peripheral fat was completely removed under binocular magnifier. The heart and 2–3 mm long aortic arch were frozen at −80 °C in OCT embedding medium for serial 10 μm-thick cryosectioning. Serial cross-sections of three valve leaflets from the aortic root were sectioned (5 sections per slide, from 200 to 850 μm from the base of the heart to the aortic arch). The glass slides were stained with Oil red O allowing visualization of neutral lipids and counterstaining with hematoxylin. Images of all stained slides were captured using a digital slide scanner. Quantitative measurement of aortic neutral lipid-stained lesions was assessed using image analysis software ‘Nanozoomer Digital Pathology.View2 software U12388-02’ allowing a morphometric analysis. Results were expressed in percentage of total aortic surface as the average of neutral lipid area for each distance from the heart, as previously described [13].

### 2.12. Statistical Analysis

Values were expressed as the mean ± standard error of mean (SEM). Statistical analyses were performed using one-way analysis of variance (ANOVA), or Kruskal–Wallis tests and subsequent Bonferroni/Sidak multiple comparisons test to estimate the effect of carrot supplementation. *p*-values lower than 0.05 was considered statistically significant. All analyses were calculated with GraphPad Prism 6 software (GraphPad Software Inc., San Diego, CA, USA).

## 3. Results

### 3.1. Carrot Supplementation Effects on Body Weight Gain

**Figure 1:**
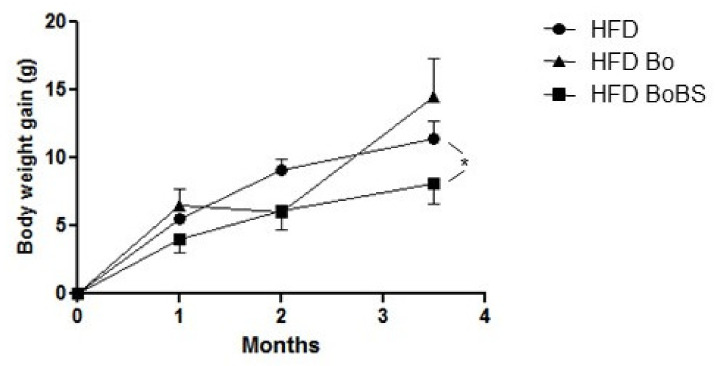
Carrot supplementation effects on body weight gain of ApoE^−/−^ mice. Time course of body weight gain in mice fed with HFD (black circle, *n* = 8; 4 males and 4 females) or HFD containing Bolero under standard condition (Bo, black triangle, *n* = 7; 5 males and 2 females) or Bolero under biotic stress condition (BoBS, black square, *n* = 6; 4 males and 2 females) for 14 weeks. Data were given as mean ± SEM. * *p* < 0.05 vs., HFD group.

Throughout the experiment, HFD induced a time-dependent body weight gain in the three different groups. The mice fed with a BoBS enriched diet displayed a significantly reduced weight gain compared to the control, while no changes were observed in Bo fed mice (Figure 1).

### 3.2. Effects of Carrot Supplementation on Hepatic Antioxidant Enzymes

**Figure 2:**
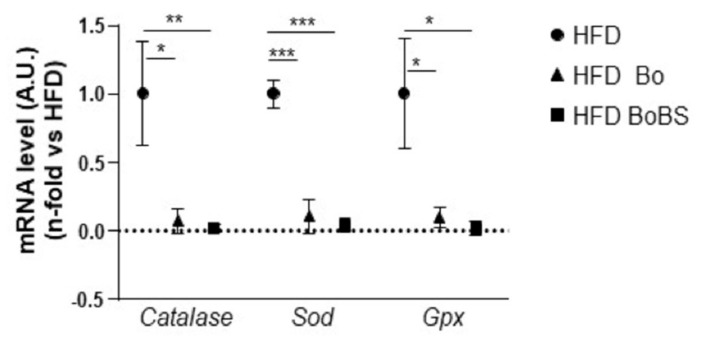
Effect of HFD (black circle, *n* = 5, samples from 5 males) or HFD containing Bolero under standard condition (Bo, black triangle, *n* = 6, samples from 6 males) or Bolero under biotic stress condition (BoBS, black square; *n* = 6, samples from 6 males) for 14 weeks on liver oxidative stress in ApoE^−/−^ mice. Hepatic relative mRNA levels of three antioxidant enzymes: catalase, superoxide dismutase (*Sod*), and glutathione peroxidase (*Gpx*). Data were given as mean ± SEM. * *p* < 0.05, ** *p* < 0.01, *** *p* < 0.001 vs., HFD group.

A 14-week supplementation with Bo and BoBS decreased the relative mRNA levels of antioxidant genes such as catalase, superoxide dismutase (*Sod*), and glutathione peroxidase (*Gpx*) compared to the HFD control group (Figure 2).

### 3.3. Carrot Supplementation Effects on Plasma Glucido-Lipidic Parameters

As previously described and expected, mice fed with HFD exhibited higher levels of plasma triglycerides (TG) compared to standard diet fed mice [8]. Interestingly, both Bo and BoBS supplementations significantly reduced circulating TG levels compared to the HFD control group (Table 2). Under our experimental conditions, none of the other analyzed parameters including glucose, insulin, cholesterol, or HDL/LDL were significantly modified by Bo or BoBS compared to HFD.

### 3.4. Effects of Carrot on Hepatic Function and Structure

#### 3.4.1. Triglycerides Hepatic Secretion

**Figure 3:**
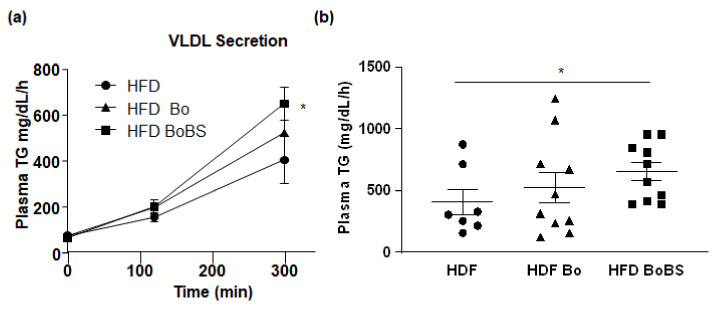
Effect of diet and carrot supplementation on very low-density-lipoprotein (VLDL) TG secretion after Triton WR-1339 injection (**a**), VLDL TG secretion rate assessed 3 h after lipoprotein lipase inhibition by Triton WR-1339 injection (**b**) in mice receiving HFD (black circle, *n* = 7, samples from 7 males), or HFD containing Bolero under standard condition (HFD Bo; black triangle, *n* = 10, samples from 10 males) or Bolero under biotic stress condition (HFD BoBS, black square, *n* = 10, samples from 10 males) for 14 weeks. Data were given as mean ± SEM. * *p* < 0.05 vs., HFD group.

To evaluate the capacity of liver to accumulate lipids, we measured the rate of hepatic VLDL TG secretion in the three groups after intraperitoneal injection of a lipoprotein lipase inhibitor. The hepatic TG secretion rate was significantly elevated in HFD mice supplemented with BoBS (648.5 ± 73.28 mg/dL/h; *p* < 0.05) compared to HFD group (404.5 ± 104 mg/dL/h) (Figure 3a,b).

#### 3.4.2. Steatosis and Fibrosis Quantification

**Figure 4:**
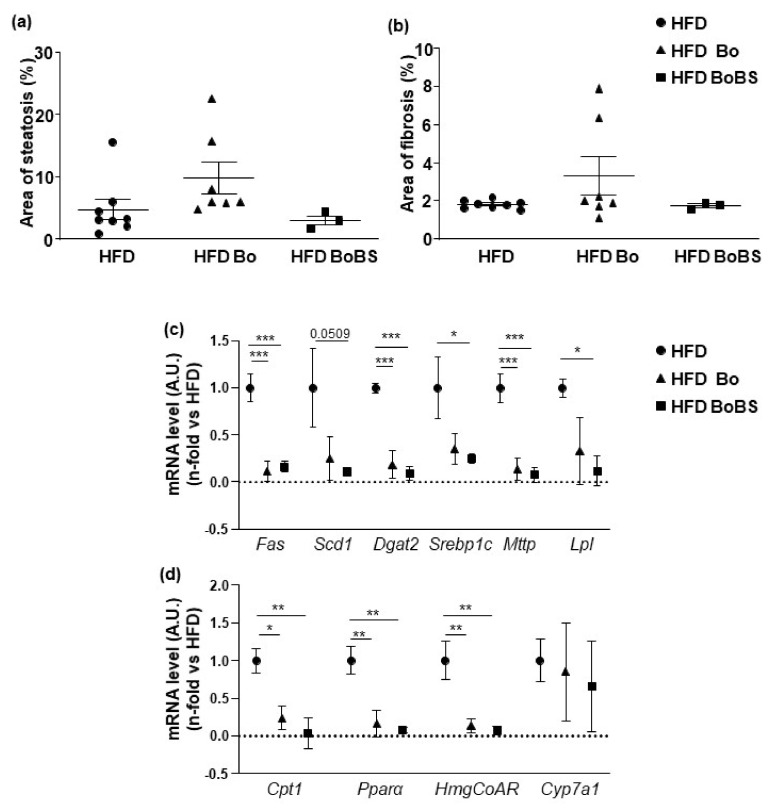
Effect of carrot supplementation on liver steatosis (**a**) and fibrosis (**b**) in ApoE^−/−^ mice fed with HFD (black circle, *n* = 8; samples from 4 males and 4 females), or HFD containing Bolero (black triangle and black square for HFD supplemented with Bolero under standard condition (Bo; *n* = 7; samples from 5 males and 2 females) or under biotic stress condition (BoBS; *n* = 3; samples from 3 males). (**c**) Relative gene expression of hepatic lipogenic enzyme (*Fas*; *Scd1*; *Dgat2*; *Srebp1c*; *Mttp*; and *Lpl*) (for HFD group *n* = 5, samples from 5 males; for HFD Bo group *n* = 6, samples from 6 males; for HFD BS group *n* = 6, samples from 6 males). (**d**) Relative hepatic gene expression of *Cpt1*; *Pparα*, *HmgCoAR*, and *Cyp7a1*. Data were given as mean ± SEM. * *p* < 0.05, ** *p* < 0.01, *** *p* < 0.001 vs., HFD group.

None of Bo or BoBS enriched diet induced improvement in hepatic steatosis and fibrosis induced by HFD (Figure 4a,b).

The mRNA levels of genes involved in lipid metabolism (fatty acid synthase (*Fas*), lipogenic stearoyl-coenzyme A desaturase-1 (*Scd1*), diacylglycerol O-acyltransferase 2 (*Dgat 2*), sterol regulatory element-binding transcription factor 1 (*Srebp1c*), microsomal TG transfer protein (*Mttp*), lipoprotein lipase (*Lpl*)), in lipid oxidation (carnitine palmitoyltransferase-1A (*Cpt1*), peroxisome proliferator activated receptor alpha (*Pparα*)) and in cholesterol synthesis (hydroxymethylglutaryl-Coenzyme A reductase, (*HmgCoAR*)) were analyzed after the 14-weeks treatment. Interestingly, both Bo and BoBS induced a decrease of all those mRNA levels (Figure 4c,d).

To be noted, the relative expression of the cytochrome P450 family 7 subfamily A member 1 (*Cyp7a1*), implicated in bile acid synthesis, was not modified versus control.

### 3.5. Effects of Carrot Supplementation on Short Chain Fatty Acid Composition

**Figure 5:**
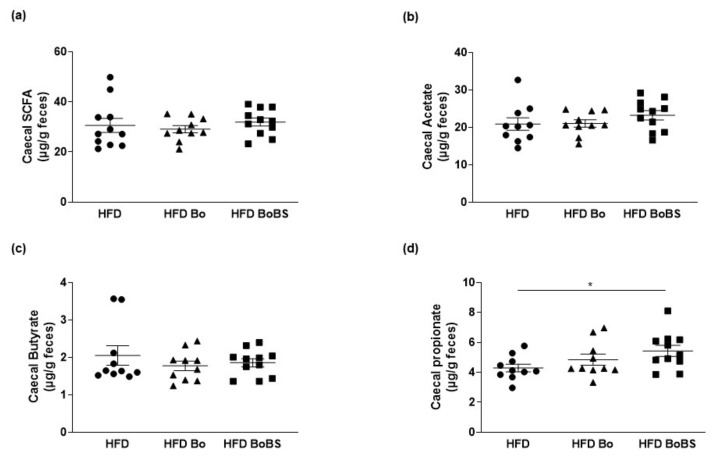
Cecal short chain fatty acids (SCFA) analysis and quantification in ApoE^−/−^ mice. Total cecal SCFA (**a**), acetate (**b**), butyrate (**c**), propionate (**d**) content in mice receiving HFD (black circle, *n* = 11; samples from 11 males) or HFD containing Bolero under standard condition (Bo, black triangle, *n* = 10; samples from 10 males) or Bolero under biotic stress condition (BoBS, black square, *n* = 11; samples from 11 males) throughout the 14 weeks of diet. Data were given as mean ± SEM. * *p* < 0.05 vs., HFD group.

The cecum is the main site of bacterial fermentation in mice. The total cecal content of butyrate and acetate were not significantly different among the different groups. Interestingly, among SFCA quantified in this study, BoBS supplementation increased cecal content of propionate compared to HFD group (Figure 5).

### 3.6. Effects of Carrot on Heart Function

As shown in Table 3, echocardiography measurements showed that Bo supplementation did not modify structural cardiac parameters. However, it improved cardiac output. In contrast, BoBS supplementation increased left ventricular end-systolic diameter (LVESD), left ventricular end-diastolic dimension (LVEDD), left ventricular end-systolic volume (LVESV), left ventricular end-diastolic volume (LVEDV), without modification of functional parameters.

### 3.7. Effects of Carrot on Blood Pressure and Heart Rate

**Figure 6:**
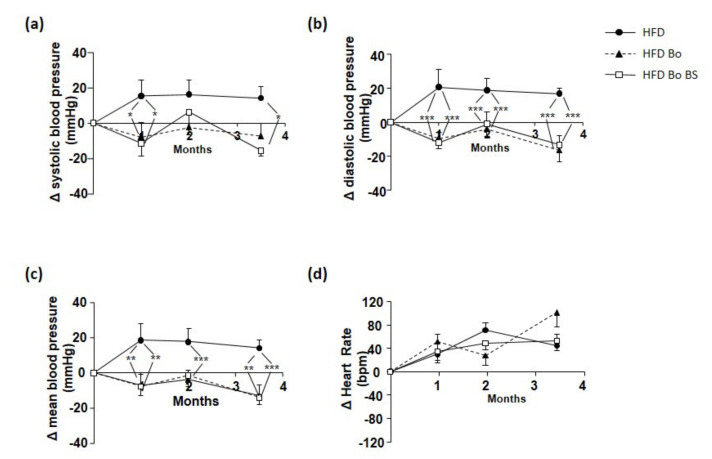
Carrot supplementation effects on hemodynamic parameters of ApoE^−/−^ mice. Systolic (**a**), diastolic (**b**), mean (**c**) pressure, and heart rate (**d**) variations in mice receiving HFD (black circle, *n* = 8; 4 males and 4 females) or HFD supplemented with Bolero under standard condition (Bo, black triangle; *n* = 7; 5 males and 2 females) or Bolero under biotic stress condition (BoBS, black square; *n* = 6; 4 males and 2 females) throughout the 14 weeks of diet. Data were given as mean ± SEM. * *p* < 0.05, ** *p* < 0.01, *** *p* < 0.001 vs., HFD group.

Interestingly, both Bo (during the first month) and BoBS (first and last weeks) induced a significant decrease of systolic blood pressure compared to HFD mice (Figure 6a).

The increase of diastolic and mean blood pressure induced by HFD (Figure 6b,c), was completely prevented by Bo and BoBS supplementation.

Finally, none of Bo or BoBS carrot supplementation induced significant changes in heart rate values during the 14 weeks of the study (Figure 6d).

### 3.8. Carrot Supplementation Incidence on Atherosclerotic Lesion

**Figure 7:**
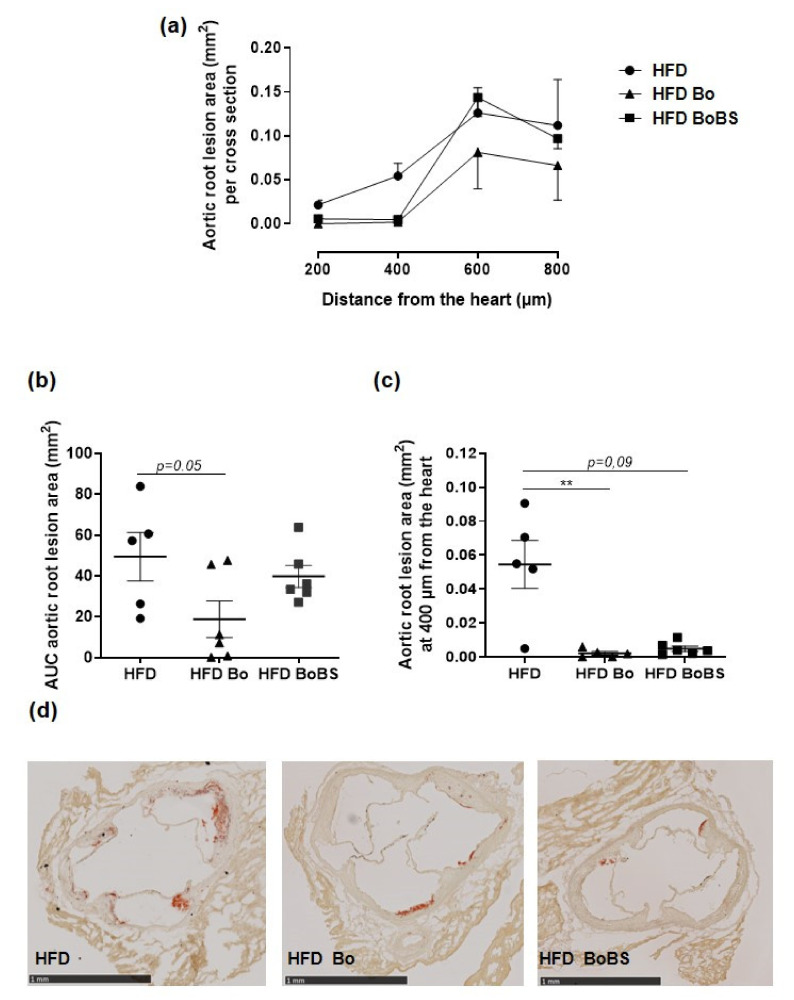
Carrot supplementation reduces atherosclerotic lesions of ApoE^−/−^ mice. Quantification of the aortic root oil red O at all distances from the heart (**a**), and subsequent calculation of AUC (**b**). Specific quantification of lesions at 400 µm from the heart (**c**), and representative images of three valve leaflets as observed at 400 µm from the heart (scale bar 1 mm) of stained aorta (**d**) from mice maintained a HFD (black circle, *n* = 5; samples from 5 males), or HFD containing Bolero under standard condition (Bo, black triangle, *n* = 6; samples from 6 males), or Bolero under biotic stress condition (BoBS, black square, *n* = 6; samples from 6 males) throughout the 14 weeks of diet. Data were expressed as mean ± SEM. ** *p* < 0.01 vs., HFD group.

The aortic root lesion was increased considering the distance from the heart to aortic arch in the HFD group (Figure 7a). Furthermore, Bo fed group displayed significantly reduced fatty streak lesions in aortic root by the calculation of area under the curve (AUC) (Figure 7a,b). Setting the analysis at 400 µm from the heart, Bo and BoBS supplementation decreased the aortic lesions size in aortic root compared to HFD group (*p* < 0.001 and *p* = 0.09 respectively) (Figure 7c,d).

## 4. Discussion

This work conducted in a mouse model of atherosclerosis shows that supplementation with carrot root from Bolero genotype grown under standard conditions or subjected to biotic stress induced by *A. dauci* inoculation counteracts most of the metabolic and cardiovascular disorders involved in atherosclerosis development. We showed that both Bo and BoBS decreased circulating TG, as the expression of genes that promote oxidative stress and hepatic *de novo* lipogenesis enzymes without modification of circulating glucose and cholesterol concentrations. In addition, BoBS supplementation decreases body weight gain, and increases VLDL secretion and cecal propionate content. Both carrot supplementations improve blood pressures. In addition, Bo supplementation increases cardiac output. Finally, Bo and BoBS reduced atherosclerotic lesions. Together, these data bring pharmacological basis of beneficial effects of both Bo carrot root varieties intake in a model of cardiometabolic diseases.

In our previous study [8], we evaluated the effect of carrot genotypes grown either under standard, water-restricted, or biotic stress induced by *A. dauci* inoculation on cellular models of cardiometabolic diseases. We found that extracts of these carrots decreased apoptosis of endothelial cells and reduced oxidative stress, lipid accumulation in hepatocytes. Also, BoBs decreases IL-6 secretion in macrophages. Thus, carrot extracts are particularly beneficial in endothelial cell, macrophages, and hepatocytes. It is known that endothelial dysfunction and inflammation play significant role in the initial step for both atherosclerosis and increased blood pressure by decreasing nitric oxide, enhancing the release of vascoconstrictor factors originating from cyclo-oxygenase metabolites in addition to endothelin. Thus, in the present study, we aimed to analyze the anti-atherosclerosis and anti-hypertensive properties of Bolero variety in two growing conditions using the ApoE^−/−^ mice fed with HFD.

In the current work, BoBS but not Bo supplementation significantly decreases the body weight gain induced by HFD. This result could be related to an increase in cecal propionate content induced by BoBS supplementation. Propionate, a major microbial fermentation metabolite present in the human gut, exerts putative beneficial health effects [14], including anorexigenic properties via the secretion of the glucagon-like peptide-1 and the activation of two orphan G protein coupled receptors (GPRs), GPR41 and GPR43 [15,16]. Propionate is a potentially interesting metabolite for preventing obesity and diabetes by regulating intestinal hormones and reducing food intake in rodent models of diet-induced obesity [17]. While not significantly different, Bo and BoBS tended to decrease *Scd1* gene expression, which might be partially involved in the bodyweight decrease observed at least for BoBS group. Indeed, mice with a low *Scd1* gene expression display enhanced energy expenditure, decreased body adiposity, increased insulin sensitivity, and are resistant to diet-induced obesity [18].

As expected, carrot supplementation modulates the oxidative stress status. Indeed, both supplementations, Bo and BoBS, induced a decrease of the relative expression of three hepatic antioxidant enzymes: catalase, superoxide dismutase (*Sod*), and glutathione peroxidase (*Gpx*). These effects are in accordance with our previous in vitro results collected in hepatocytes. We found that both Bo and BoBS protected HepG2 cells from oxidative stress induced by hydrogen peroxide [8]. We previously described that Bo variety accumulates large quantities of polyphenols and carotenoids with antioxidant property compared to other studied varieties including Karotan, Presto, Deep Purple Kintoki, and Blanche des Vosges [8]. It is noteworthy that the synergistic effects of these compounds induced a sparing in endogenous anti-oxidative defenses as shown by decreased catalase, SOD, and Gpx activities in accordance with the report of Vijayakumar et al. [19].

With regard to the cardiac parameters, Bo increases cardiac output without affecting the structure parameters. Although BoBS supplementation increases left ventricular structure, it does not affect left ventricular weight (data not shown) and does not reduce cardiac function even if it tends to increase cardiac output. Differential data on the effect of carrot on the heart have been reported. In rat models of type 1 diabetes, carrot supplementation was reported to have no effect on cardiac abnormalities [20]. Conversely, in a rat model of metabolic syndrome, purple carrot juice supplementation but not β-carotene reduced left ventricular stiffness, improved cardiac functions and structure [6]. These authors attributed the beneficial effect of Purple carrot juice to its anthocyanin content. In our previous study, we have found that Bo is among the carrot varieties that possess the highest levels of polyphenols. Thus, one can hypothesize that these polyphenols participate in the improvement of cardiac output measured at least with Bo supplementation.

The most novel findings are that both Bo and BoBS reduced significantly systolic, diastolic, and mean arterial blood pressure without affecting heart rate. These findings demonstrated an antihypertensive effect of Bo in a mouse model of atherosclerosis independently of the culture method in accordance with previously reported results: reduced oxidative stress in endothelial and smooth muscle cells in vitro treated with Bo [8]. It is likely that both carotenoids and polyphenols contents in carrot have decreasing oxidative stress properties in our mice model of atherosclerosis by their ability, not only to reduce reactive oxygen species production, but also by increasing both nitric oxide (NO) availability and production [3,4,21,22,23]. Bo is rich in α- and β-carotens, phenolic acids, and flavonoids such as anthocyanins [8,24]. These substances are known to reduce oxidative stress and also to increase NO production and sirtuin activation that trigger health benefit in cardiovascular disturbances including hypertension. These data provide pre-clinical basis of the report showing that carrot belongs to vegetables associated with a lower risk of hypertension [25]. Indeed, a human study reported that carrot juice contributes to a 5% reduction in systolic blood pressure [7], that is consistent with the National Heart, Lung, and Blood Institute’s recommendations to increase intake of vegetables as part of a dietary pattern [26]. Furthermore, Li et al. showed that carotenoids consumption is inversely associated with hypertension with a significant effect of lower risk of hypertension [27].

Supplementation with BoBS modulated lipid metabolism by reducing circulating TG and the expression of genes that promote hepatic *de novo* lipogenesis, these results being in contradiction with the increased hepatic VLDL secretion. This apparent discrepancy could be explained by a higher VLDL catabolism which was not analyzed in the present study. The increased VLDL secretion could be beneficial, when their catabolism is also elevated, to contract the development of steatosis and fibrosis although we did not observe any effect on hepatic structure and lipid content. Controversial data have been reported in the literature concerning these parameters. Carrot diet positively affects lipid metabolism in rats [5]. It was also shown that mice fed with cholesterol-supplemented diets and lyophilized carrot for 4-week presented reduced cholesterol and triglycerides levels in plasma and liver [28]. However, carrot juice supplementation during three months in adult dyslipidemic subjects did not modify plasma cholesterol, triglycerides, LDL, or HDL [7]. These differences in the results might be due to species, animal models, varieties of carrot, and the use of whole carrot *versus* carrot juice.

The antihypertensive activity of carrot concomitant with its lipid lowering capacity and anti-oxidative stress described in this report could contribute to the reduction of atherosclerotic lesion. Indeed, particularly Bo and to a lesser extent BoBS reduced the area of aortic lesion in HFD mice. Moreover, although Bo and BoBS did not affect LDL levels in this study, one can advance the hypothesis that they may rather reduce the oxidized LDL. Oxidized LDL in the subendothelial space participate in endothelial dysfunction by increasing the expression of adhesion molecules leading to monocyte infiltration and formation of foam cells, leading to atherosclerosis development. In accordance with the present work, Wang et al. [29] found that carotenoid supplementation lowered intima–media thickness in aged Chinese. Together, these effects of carotenoids suggest anti-atherosclerotic carrot properties. Polyphenols contained in carrot might also participate in anti-atherosclerotic effects obtained in the present study as reported by Luo et al. (2020), showing that quercetin inhibits endothelial dysfunction and atherosclerosis in ApoE^−/−^ mice [30].

## 5. Conclusions

In conclusion, we demonstrate that dietary Bo and BoBS exhibit antihypertensive and anti-atherosclerotic properties independently of growing conditions in a mouse model of atherosclerosis. Therefore, Bo constitutes dietary natural compounds for human nutrition and health and may represents a novel category of dietary products for the prevention or the management of vascular and metabolic diseases.

This study presents strengths and limitations. The use of male and female mice for some measurements may appear as a limitation. Despite this limitation, overall results obtained in the two studies conducted in two different laboratories converge towards a protective effect of carrots on cardiovascular risk which constitutes a strong point of our report. Further preclinical and clinical studies—focusing on the compound(s) of the BO carrot responsible for these effects—are needed.

## Figures and Tables

**Table 1:** Nutritional composition and energy content of diets.

Diet	High Fat Diet	High Fat Diet + Bolero (Bo or BoBS)
Composition, g/kg		
Bo or BoBS	without	25
Sucrose	340	340
Dairy butter	200	200
Casein	180.5	180.5
Pregelatinized cornstarch	145	145
Premixture of minerals	70	70
Crude cellulose	50	46.77
Premixture of vitamins	10	10
DL-methionine	3	3
Cholesterol	1.43	1.43
Energy, %		
Protein	17.7	17.7
Fat	41.7	41.7
Carbohydrate	40.6	40.6

**Table 2:** Carrot supplementation impacts on plasma glucose and lipid parameters.

	HFD	HFD Bo	HFD BoBS
Glucose (g/L)	1.81 ± 0.34	1.23 ± 0.22	0.89 ± 0.21
Insulin (g/L)	0.58 ± 0.03	0.86 ± 0.15	0.83 ± 0.09
Triglycerides (g/L)	1.34 ± 0.17	0.77 ± 0.14 *	0.44 ± 0.06 ***
Cholesterol (g/L)	7.70 ± 2.25	7.41 ± 1.06	6.37 ± 1.14
HDL/LDL	0.09 ± 0.02	0.24 ± 0.06	0.19 ± 0.05

Circulating levels of glucose, insulin, triglycerides, total cholesterol, and HDL/LDL ratio evaluated in plasma from fasted mice receiving HFD (*n* = 8; samples from 4 males and 4 females) or HFD containing Bolero under standard condition (Bo; *n* = 7; samples from 5 males and 2 females) or Bolero under biotic stress condition (BoBS; *n* = 6; samples from 4 males and 2 females) for 14 weeks. Data were given as mean ± SEM. * *p* < 0.05, *** *p* < 0.001 vs., HFD.

**Table 3:** Heart function throughout the diet.

	HFD	HFD Bo	HFD BoBS
LVESD (mm)	2.3 ± 0.1	2.2 ± 0.1	2.8 ± 0.1 *
LVEDD (mm)	3.6 ± 0.1	3.5 ± 0.1	4 ± 0.1 *
LVESV (mL)	19.4 ± 2.2	16.2 ± 1.4	29.8 ± 3.7 *
LVEDV (mL)	55.3 ± 2.4	52.5 ± 4	73 ± 6 *
Stroke volume (mL)	35.9 ± 2.4	36.3 ± 3.4	43.2 ± 3
Ejection fraction (%)	65.1 ± 3.6	68.9 ± 2	59 ± 5
Shortening fraction (%)	35.5 ± 2.7	38 ± 1.6	31 ± 2.1
Cardiac output (mL/min)	17.4 ± 2.5	28.5 ± 4 **	25.5 ± 5

The table shows the LVESD, LVEDD, LVESV, LVEDV, stroke volume, ejection fraction, shortening fraction, and cardiac output of mice fed with HFD (*n* = 8; 4 males and 4 females), or HFD supplemented with Bolero under standard condition (Bo; *n* = 7; 5 males and 2 females) or under biotic stress condition (BoBS; *n* = 3; 3 males) for 14 weeks. Data were given as mean ± SEM. * *p* < 0.05, ** *p* < 0.01 vs., HFD.

## Data Availability

All animal protocols were approved by the local ethics committee “Comité d’éthique en experimentation animale Pays de la Loire”; (Project No. Apafis#1687-2015060312546655 and Apafis#320-2015031314466612_v2) and realized in agreement with the guidelines and authorization with French Ministry of Agriculture regulations according to the European Union regulations (2010/63/EU).
